# Prevalence of Back and Neck Pain Among Surgeons Regardless of Their Specialties in Saudi Arabia

**DOI:** 10.7759/cureus.49421

**Published:** 2023-11-26

**Authors:** Lujain Alshareef, Fatimah Al Luhaybi, Rawan S Alsamli, Amirah Alsulami, Ghania Alfahmi, Wefag A Mohamedelhussein, Alhassan Almaghrabi

**Affiliations:** 1 College of Medicine and Surgery, Umm Al-Qura University, Makkah, SAU; 2 Faculty of Medicine, International University of Africa, Khartoum, SDN; 3 Department of General Surgery, Al Noor Specialist Hospital, Makkah, SAU

**Keywords:** saudi arabia, neck pain, back pain, prevalence, surgeons, pain, musculoskeletal

## Abstract

Background: Musculoskeletal disorders are the second-most common complaint among surgeons.

Objectives: This study aimed to determine the prevalence of back and neck pain among Saudi surgeons of all specialties.

Materials and methods: A descriptive, cross-sectional study was used to collect data from 195 surgeons of different specialties in Saudi Arabia. The data were collected using a self-administered questionnaire to measure back pain, neck pain, and overall musculoskeletal pain (MSP).

Results: Surgeons have a high level of neck and back pain. The prevalence of back, neck, and shoulder pain among the studied surgeons was 68.2%, 56.9%, and 46.2%, respectively, while the overall prevalence of MSP was 87.2%. MSP was significantly (p <.05) higher among male surgeons, in those who experienced fatigue only on long days, and in those who reported that the cause of fatigue was laparoscopic procedures.

Conclusion: Musculoskeletal symptoms are highly prevalent among surgeons in Saudi Arabia. Therefore, more studies should be conducted to assess and identify all the potential risk factors involved as well as ergonomic strategies to reduce the prevalence of MSP among surgeons, improve their quality of life, and avoid further complications.

## Introduction

​​Musculoskeletal disorders (MSDs) are the second-most common complaint among surgeons [1]. According to research, pain is the primary reason for more than 50% of visits to the emergency room and 30% of visits to the family doctor. The prevalence of chronic pain has been found to range from 12 to 80% of the population, according to a number of epidemiological studies [2]. The most common type of pain is back and neck pain. There is still a lack of studies that include surgeons from all surgical specialties across Saudi Arabia, so the goal of this study was to assess the prevalence of back and neck pain among surgeons in Saudi Arabia, regardless of their specialties [1].

Back disorders have become the predominant reason for disability in those younger than 45 [3]. MSDs pose a challenge and have emerged as a significant issue in our society’s interconnected workplaces [1]. Work-related MSDs (WMSDs) are a group of symptoms that are exacerbated by working and manifest as persistent pain, soreness, discomfort, and impairment [1]. They have been recognized to be associated with various occupational risk factors, as well as work position, posture, physical force, vibration, and movement [4]. Musculoskeletal (MSK) problems are caused by a combination of ergonomic, physical, and emotional factors [5].

According to research, MSK issues are the second-most common issue surgeons face at work, after mental stress [6]. The most common types of pain were neck and back pain, which affected 90% of surgeons in the UK [6]. Surgery can be physically taxing, especially with the rise of laparoscopic procedures that require more rigid body positions [7,8].

Advancements in laparoscopic surgery have primarily focused on enhancing patient benefits. However, compared with open surgery, laparoscopic surgery imposes greater ergonomic constraints on surgeons. Recent reports indicate a 73% to 88% prevalence of physical complaints among laparoscopic surgeons, which is greater than in the general working population, supporting the need to address the surgeons' physical health [9].

The physical impact of laparoscopic procedures on the surgeon is a major complication today [10]. Avoiding static loads, reducing sedentary working postures, and maintaining a frontal neck position of less than 15° are all recommendations from the National Institute for Occupational Safety and Health [11]. Despite these recommendations, documented poses are primarily influenced by surgeons’ training and one’s personal decision to disregard these recommendations [12]. According to a study conducted in western New York, 77% of respondents reported back pain, while 74% reported neck pain [13]. The same study found that 16 surgeons had received musculoskeletal pain (MSP) treatment in the past or currently, and 14 surgeons said that their pain had forced them to change their practice [13]. Another study, in Jeddah, Saudi Arabia, discovered that the majority of respondents (80%) experienced MSK symptoms associated with surgery, with the neck and back being the most affected body parts [11].

A significantly higher proportion of respondents with MSK symptoms (28.1%) had been practicing for 5 to 10 years [11]. Although MSK symptoms are common among Saudi surgeons [11], there is still a scarcity of studies that include surgeons from all surgical specialties in Saudi Arabia. Thus, the purpose of this study was to determine the prevalence of back and neck pain among Saudi surgeons of all specialties.

## Materials and methods

Study design, setting, and time

A cross-sectional study was done in Saudi Arabia in the period from April to June 2023. 

Study participants 

The inclusion criteria were all surgeons in different specialties (residents, specialists, consultants) who consented after the study’s purpose and objectives were explained to them, and the exclusion criteria were medical interns and physicians other than surgeons.

Data collection

An online, predesigned questionnaire was prepared in a Google Form and directed to all surgeons in Saudi Arabia regardless of their specialties during the study period. The questionnaire was discussed and approved by qualified surgeons. The questionnaire comprised two sections. The first section addressed the surgeons’ general information, such as age; gender; specialty; number of years in practice; previous neck, back, or shoulder pain; and contributing factors to that pain. The second section included a modified version of a questionnaire from a previous study with similar objectives [14] that contained questions about surgical practice, such as the mix of open and laparoscopic procedures; fatigue and its frequency and reasons; changes in laparoscopic procedures that may reduce pain and fatigue; and attitude toward robotic surgery. 

Ethical considerations 

All the respondents received electronic links accompanied by the objectives of the survey and a request to participate voluntarily, and ethical approval was obtained from the Institutional Research Board of Um-Al Qura University (UQU), Makkah, Saudi Arabia (approval no. HAPO-02-K-012-2023-02-1470). 

Data analysis

The data were analyzed using IBM SPSS Statistics for Windows, Version 26 (Released 2019; IBM Corp., Armonk, New York, United States). To assess the relationship between the variables, the chi-squared test (χ2) was applied to qualitative data, which were expressed as numbers and percentages. A p-value of less than .05 was considered statistically significant.

## Results

Table 1 shows that the largest proportion of the participants (36.4%) had an age ranging from 31 to 35 years, 66.2% were males, and 42.6% had a general surgery specialty. 

**Table 1:** Distribution of the studied surgeons according to their demographics and specialty (N = 195)

Variable	No. (%)
		Age	
26–30	1 (0.5)
31–35	71 (36.4)
36–40	43 (22.1)
41–45	40 (20.5)
46–50	13 (6.7)
51–55	9 (4.6)
56–60	6 (3.1)
61–65	6 (3.1)
>65	6 (3.1)
Gender	
Female	66 (33.8)
Male	129 (66.2)
Specialty	
Cardiac surgery	6 (3.1)
Colorectal	1 (0.5)
ENT	13 (6.7)
General surgery	83 (42.6)
Hepato-pancreaticobiliary surgery	1 (0.5)
Maxillofacial surgery	1 (0.5)
Neurosurgery	11 (5.6)
Obstetrics and gynecology	25 (12.8)
Orthopedics	17 (8.7)
Pediatric surgery	8 (4.1)
Plastic surgery	7 (3.6)
Thoracic surgery	4 (2.1)
Urology	15 (7.7)
Vascular surgery	3 (1.5)

Of the participants, 38.5% had a work experience of 0-5 years, and 74.4% worked on both laparoscopic and open procedures (Table 2). Most of them (65.6%) had been taught to maintain proper posture during surgical procedures when trained as medical students or residents, and 70.8% considered their posture while operating. Of those using robotic surgeries, the most commonly mentioned benefit was being more comfortable for the surgeon (37.9%).

**Table 2:** Distribution of the studied surgeons according to work experience, type of surgery, maintaining proper posture during surgical procedures, and benefits of robotic surgery (N = 195)

Variable	No. (%)
Work experience (years)	
0–5	75 (38.5)
6–10	53 (27.2)
11–15	36 (18.5)
16–20	14 (7.2)
21–25	9 (4.6)
>25	8 (4.1)
Type of surgery	
Laparoscopic procedures	11 (5.6)
Open procedures	39 (20.0)
Both	145 (74.4)
When being trained as a medical student or resident, were you taught to keep proper posture during surgical procedures?	
No	67 (34.4)
Yes	128 (65.6)
Do you consider your posture while operating?	
No	57 (29.2)
Yes	138 (70.8)
If you currently perform robotic surgery, check all benefits that you feel apply:	
Decreased recovery time	40 (20.5)
More comfortable for the surgeon	74 (37.9)
Increased quality of surgery	63 (32.3)
Increased range of surgical candidacy (i.e., can be performed on morbidly obese patients)	31 (15.9)
Increased surgeries from referrals	15 (7.7)

**Figure 1:**
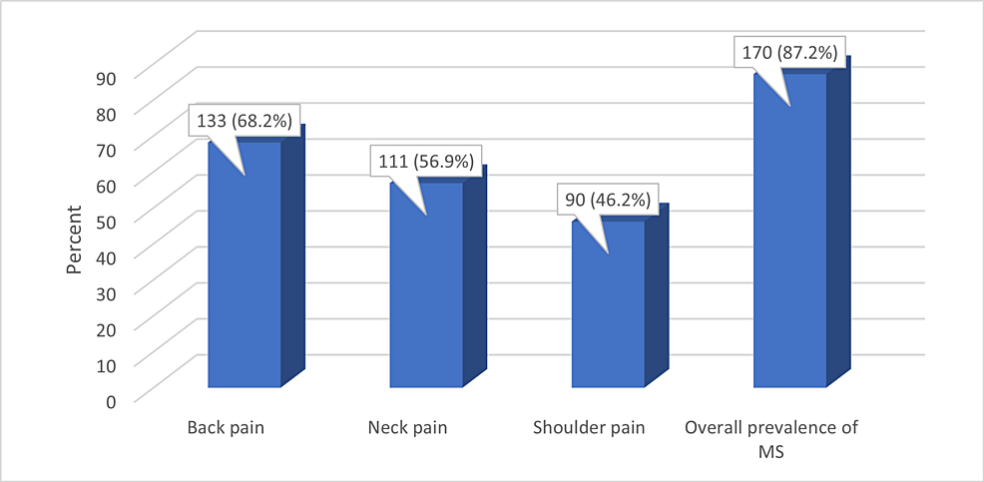
Percentage distribution of the participants according to the prevalence of back, neck, and shoulder pain and overall prevalence of MSP MSP: Musculoskeletal pain

Figure 1 illustrates that the prevalence of back, neck, and shoulder pain among the studied surgeons was 68.2%, 56.9%, and 46.2%, respectively, while the overall prevalence of MSP was 87.2%.

Table 3 demonstrates that, among the 145 surgeons who performed both open and laparoscopic procedures, 38.6% had an approximate percentage of 50% open to 50% laparoscopic. Among the 156 surgeons who performed laparoscopic procedures, 61.5% incorporated table height adjustment into the majority of their procedures. A majority of the participants (82.1%) experienced fatigue during procedures. Of the participants, 64.5% experienced fatigue only on long days, and the most common cause was long surgery times 88.1%.

**Table 3:** Distribution of data about patterns of performed surgeries, experiencing fatigue, and its frequency and causes (N = 195) MSP: Musculoskeletal pain

Variable	No. (%)
If you perform both open and laparoscopic procedures, what is the approximate percentage of open to laparoscopic? (n = 145)	
100% lap	2 (1.3)
100% open	13 (8.9)
50% lap	56 (38.6)
75% lap	30 (20.6)
75% open	44 (30.6)
If you perform laparoscopic procedures, what do you incorporate into the majority of your procedures? (n = 156)	
Adjustable monitors 4.07	66 (42.3)
Table height adjustment	96 (61.5)
Stools to sit while operating	37 (23.7)
Moments to stretch in long procedures	55 (35.2)
Do you ever experience fatigue during procedures?	
No	35 (17.9)
Yes	160 (82.1)
How often do you experience fatigue? (n = 160)	
1–2/week	10 (6.2)
Most days in surgery	47 (29.3)
Only on long days	103 (64.5)
Causes of fatigue (n = 160)	
Long surgery times	141 (88.1)
Decreased sleep	89 (55.6)
Stress from work	100 (62.5)
Stress from outside work	40 (25.0)
Outside hobbies	14 (8.7)
Open procedures	48 (30.0)
Laparoscopic procedures	44 (27.5)
Participants were distributed based on the prevalence of back, neck, and shoulder pain as well as the total prevalence of MSP.	No.(%)
Back pain	68.2
Neck pain	56.9
Shoulder pain	46.2
Overall prevalence of MSP	87.2

Table 4 shows that, among the surgeons who reported MSP (n = 170), the most common cause of pain was an outside hobby that put them at risk of excess use or strain on those parts of the body (33.3%), and, for 44.7%, the pain was experienced 0-2 times weekly. Only 38.2% reported that the pain made them seek medical attention. The most commonly used treatment for pain was non-steroidal anti-inflammatory drugs (NSAIDs).

**Table 4:** Pain characteristics among surgeons who reported having any MSP (n = 170) MSP: Musculoskeletal pain: NSAIDs: non-steroidal anti-inflammatory drugs

Variable	No. (%)
Causes of pain	
Previous traumatic injury to that part of the body	34 (17.4)
Medical condition that predisposes you to pain in that part(s) of the body	52 (26.7)
Outside hobby that puts you at risk for excess use or strain on that part(s) of the body	65 (33.3)
Long procedures/wrong posture	6 (3.1)
None	13 (7.6)
How often does the pain occur?	
0–2 times/month	57 (33.5)
0–2 times/week	76 (44.7)
2–4 times/week	26 (15.4)
Daily	11 (6.4)
Has the pain caused you to seek medical attention?	
No	105 (61.8)
Yes	65 (38.2)
Have you used or had any of the following for treatment?	
NSAIDs	107 (62.9)
Physical therapy	92 (54.1)
Prescribed medication	32 (18.8)

Tables 5, 6 show that the prevalence of MSP was significantly higher among male surgeons, those who experienced fatigue only on long days, and those who reported that the cause of fatigue was laparoscopic procedures (p <.05). 

**Table 5:** Relationship between MSP prevalence and surgeons’ demographics, specialty, work experience, type of surgery, proper posture during surgical procedures, and benefits of robotic surgery (N = 195) MSP: Musculoskeletal pain

Variable	MSP		χ2	p-value
	Yes	No		
	No. (%)	No. (%)		
Age (years)				
≤45	145 (85.3)	22 (88.0)	0.13	0.719
>45	25 (14.7)	3 (12.0)		
Gender				
Female	62 (36.5)	4 (16.0)	4.07	0.043
Male	108 (63.5)	21 (84.0)		
Specialty				
Cardiac surgery	6 (3.5)	0 (0.0)	15.28	0.296
Colorectal	1 (0.6)	0 (0.0)		
ENT	10 (5.9)	3 (12.0)		
General surgery	72 (42.4)	11 (44.0)		
Hepato-pancreaticobiliary surgery	1 (0.6)	0 (0.0)		
Maxillofacial surgery	1 (0.6)	0 (0.0)		
Neurosurgery	10 (5.9)	1 (4.0)		
Obstetrics and gynecology	23 (13.5)	2 (8.0)		
Orthopedics	14 (8.2)	3 (12.0)		
Pediatric surgery	8 (4.7)	0 (0.0)		
Plastic surgery	5 (2.9)	2 (8.0)		
Thoracic surgery	4 (2.4)	0 (0.0)		
Urology	14 (8.2)	1 (4.0)		
Vascular surgery	1 (0.6)	2 (8.0)		
Work experience (years)				
0–5	61 (35.9)	14 (56.0)	4.13	0.53
6–10	49 (28.8)	4 (16.0)		
11–15	32 (18.8)	4 (16.0)		
16–20	13 (7.6)	1 (0.4)		
21–25	8 (4.7)	1 (0.4)		
>25	7 (4.1)			
Type of surgery				
Laparoscopic procedures	10 (5.9)	1 (4.0)	4.6	0.1
Open procedures	30 (17.6)	9 (36.0)		
Both	130 (76.5)	15 (60.0)		
When being trained as a medical student or resident, were you taught to keep proper posture during surgical procedures?				
No	56 (32.9)	11 (44)	1.18	0.277
Yes	114 (67.1)	14 (56)		
Do you consider your posture while operating?				
No	49 (28.8)	8 (32)	0.1	0.744
Yes	121 (71.2)	17 (68)		

**Table 6:** Relationship between MSP prevalence and percentage of open to laparoscopic surgeries and experiencing fatigue and its pattern (N = 195) MSP: Musculoskeletal pain

Variable	MSP		χ2	p-value
	Yes	No		
	No. (%)	No. (%)		
If you perform both open and laparoscopic procedures, what is the approximate percentage of open to laparoscopic? (n = 145)				
100% lap	2 (1.2)	0 (0.0)	3.99	0.551
100% open	11 (6.5)	2 (8.0)		
50% lap	51 (30.0)	5 (20.0)		
75% lap	26 (15.3)	4 (16.0)		
75% open	40 (23.5)	4 (16.0)		
Do you ever experience fatigue during procedures?				
No	27 (15.9)	8 (32.0)	3.84	0.05
Yes	143 (84.1)	17 (68.0)		
How often do you experience fatigue? (n = 160)				
1–2/week	9 (5.3)	1 (4.0)	8.31	0.04
Most days in surgery	46 (27.1)	1 (4.0)		
Only on long days	88 (51.8)	15 (60.0)		
Causes of fatigue				
Long surgery times	124 (72.9)	17 (68.0)	0.26	0.606
Decreased sleep	79 (46.5)	11 (44.0)	0.05	0.817
Stress from work	91 (53.5)	9 (36.0)	2.68	0.102
Stress from outside work	37 (21.9)	3 (12.0)	1.3	0.254
Outside hobbies	12 (7.1)	2 (8.0)	0.02	0.865
Open procedures	44 (25.9)	4 (16.0)	1.14	0.284
Laparoscopic procedures	43 (25.3)	1 (4.0)	5.65	0.017

## Discussion

The majority of healthcare professionals have work-related MSK issues [15]. In our study, we aimed to assess the prevalence of back and neck pain among surgeons in Saudi Arabia. In the present study, we discovered that 68.2%, 56.9%, and 46.2% of the surgeons had back, neck, and shoulder pain, respectively. According to the survey’s findings, general surgeons made up the largest proportion at 42.6%, followed by OB-GYN surgeons and orthopedic surgeons. In contrast, a previous study in Karachi indicated that orthopedic surgeons were the most prevalent, with a frequency of 30%, followed by ENT surgeons, with a frequency of 21%, and general surgeons, with a frequency of 15% [6].

In our study, there was no statistically significant association between MSP and years of experience. By contrast, a study conducted among surgeons at King Abdulaziz University Hospital in Jeddah found that surgeons who had practiced for 5-10 years had a significantly higher percentage of MSK symptoms [11].

Our result showed that 82.1 % of the participants experienced fatigue during procedures, and 38.6% of them conducted 50% laparoscopic-to-open procedure ratio, similar to a previous study ‏conducted on laparoscopic surgeons reported that 74% of surgeons complained of musculoskeletal pain and fatigue. ‏This demonstrates the increasing risk of developing work-related musculoskeletal disorders in the laparoscopic procedure [16].

Our study demonstrates that 38.6% of the 145 surgeons who conducted both open and laparoscopic operations had a roughly 50% laparoscopic-to-open procedure ratio. Furthermore, 61.5% of the 156 surgeons who conducted laparoscopic surgeries did so in the overwhelming majority of their operations. A majority of the participants (82.1%) became tired when performing operations. In 64.5% of the participants, only long days resulted in weariness, with lengthy surgical durations accounting for the majority of cases (88.1%). Among the surgeons who participated in the study, neck, shoulder, and back discomfort affected 56.9%, 46.2%, and 68.2%, respectively, while 87.2% of them reported having MSP overall.

Our results show that, among the surgeons who reported MSP (n = 170), the most common cause of pain was an outside hobby that put them at risk for excess use or strain on those parts of the body (33.3%). In comparison, a study among surgeons at a tertiary care center showed that 93.8% of the symptoms were linked to the surgical activity itself [6].

Our results indicate that the prevalence of MSP was significantly higher among those who reported that the cause of fatigue was laparoscopic surgery, similar to another study that showed that the highest proportion of respondents with pain performed laparoscopic procedures (55.4%) [11]. This further highlights the need for proper training regarding correct operating positions and for increasing awareness of the importance of physical exercise among laparoscopic surgeons.

There is also a correlation between the surgeon’s gender and the prevalence of MSP, as our results indicate that MSP prevalence was higher among male surgeons. This result was almost comparable to that of a study performed among 121 surgeons at a tertiary care center. A majority of the respondents (80%) suffered from MSK manifestations, of which 61.2% were male surgeons [11].

The results reveal an association between the duration of operations and an increase in MSP incidence among surgeons, as there is a highly significant correlation between MSP episodes with those who have long days. Another study, which was conducted in Karachi, reveals that gynecologists were less affected by MSP. Comparatively shorter working hours lead to fewer pain cases [6].

Limitations, strengths, and recommendations

The health of our surgeons and the longevity of their careers may be protected, thus enabling them to provide better care for our patients. Appropriate prospective research must be conducted in the future to look at the psychological and physical causes of pain. Also, to address campaign targets regarding WMSDs, training programs should be offered at residency which are focused on the prevention of MSK injury to reduce occupational MSK injuries and, subsequently, to enhance surgical longevity and improve the careers and lives of surgeons. 

There are several limitations to this study. This was a cross-sectional study. As a result, causal inferences may not be proven. Second, recall and desirability biases may be present due to the self-administered questionnaire that was used to collect the data. Additionally, an online survey strategy was used for this study. It was available only to those with internet access. Finally, this study was limited to a specific population.

## Conclusions

In our study, we aimed to assess the prevalence of back and neck pain among surgeons in Saudi Arabia. Our results revealed that the majority of the participants experienced MSP during their jobs regardless of their surgical specialty or the risk factors that might increase their susceptibility to experience it. Moreover, nearly half of the surgeons who conducted both open and laparoscopic operations had a roughly 50% laparoscopic-to-open procedure ratio regarding the cause of MSP. Therefore, more studies should be conducted to assess and identify all the potential risk factors involved in reducing the prevalence of MSP among surgeons to improve the outcomes and prevent further complications.
